# Complex Dynamical Behaviour in an Epidemic Model with Control

**DOI:** 10.1007/s11538-016-0217-6

**Published:** 2016-10-18

**Authors:** Martin Vyska, Christopher Gilligan

**Affiliations:** University of Cambridge, Cambridge, United Kingdom

**Keywords:** Disease dynamics modelling, Control of epidemics, Bifurcations in epidemic models

## Abstract

We analyse the dynamical behaviour of a simple, widely used model that integrates epidemiological dynamics with disease control and economic constraint on the control resources. We consider both the deterministic model and its stochastic counterpart. Despite its simplicity, the model exhibits mathematically rich dynamics, including multiple stable fixed points and stable limit cycles arising from global bifurcations. We show that the existence of the limit cycles in the deterministic model has important consequences in modelling the range of potential effects the control can have. The stochastic effects further interact with the deterministic dynamical structure by facilitating transitions between different attractors of the system. The interaction is important for the predictive power of the model and in using the model to optimize allocation when resources for control are limited. We conclude that when studying models with constrained control, special care should be given to the dynamical behaviour of the system and its interplay with stochastic effects.

## Introduction

There is increasing interest in the integration of epidemiological models of control with economic considerations (Klein et al. 2007; Geoffard and Philipson 1996). Recently, researchers have focused on models of control with economical constraints on the control resources and used optimal control theory to provide insights into optimal resource allocation strategies. These models range from allocation of treatment resources (Forster and Gilligan 2007; Goldman and Lightwood 2002) to problems of how to divide resources between treatment and detection efforts (Ndeffo Mbah and Gilligan 2010). However, in the conventional analysis the exact dynamics of the epidemiological models with constrained control have not been investigated in detail. Here by constrained control we refer to control which can be applied to some but not necessarily all the individuals in a population due to limited resources.

In this paper, we select a simple, but widely used (Rowthorn et al. 2009; Ndeffo Mbah and Gilligan 2011) epidemic model with constrained control and we examine its deterministic dynamical behaviour. We show that despite its simplicity, the model exhibits mathematically rich behaviour including stable limit cycles and their global bifurcations. The presence of limit cycles in dynamical systems has long been of interest in mathematical biosciences, particularly in ecology (Kaung and Freedman 1988; Hastings 2001; Toupo and Strogatz 2015) and epidemiology (Hethcote and Levin 1989; Wang and Ruan 2004; Jin et al. 2007). We demonstrate that the presence of limit cycles has important consequences for modelling the impacts of control. We also show that in some parts of parameter space the model exhibits counter-intuitive behaviour in which lower initial disease prevalence leads to a higher-prevalence endemic equilibrium. Whenever possible, we provide analytical conditions on the parameters of the model that give rise to the particular dynamics.

We then examine the sensitivity of the dynamical behaviour when stochasticity is introduced to the model to allow for inherent variability of the infection and recovery processes. We do this by using the Gillespie construction (Gillespie 1976) to model every event in the system as an exponential random process with rates given by the deterministic model. Thus, the stochastic effects we introduce are demographic in nature. We demonstrate that the existence of the limit cycles in the deterministic version of the model strongly impacts the behaviour of the stochastic version of the model. The stochastic fluctuations can cause transitions between different attractors of the system and in some cases can lead to extinction of the pathogen by perturbing the system onto a limit cycle which passes close to the line of zero prevalence in the phase space. Similar transitions between different attractors of the dynamical system have been previously studied in systems with seasonal forcing (Keeling et al. 2001).

Our work also demonstrates that economical constraints on control in epidemiological models can lead to the existence of weakly stable attractors and complex bifurcation dynamics. These in turn cause qualitative differences between the behaviour of the deterministic model and its stochastic counterpart. This interaction between the bifurcation dynamics and stochastic effects is important both for the predictive power of the model and in using the model to optimize resource allocation, since emergence of the limit cycles in the deterministic model causes rapid changes in the probability of eradication in the stochastic model. We conclude that when interpreting model predictions and especially when studying models with constrained control, special care should be given to the dynamical behaviour of the system and its interplay with the stochastic effects.

## Model Description

**Fig. 1:**
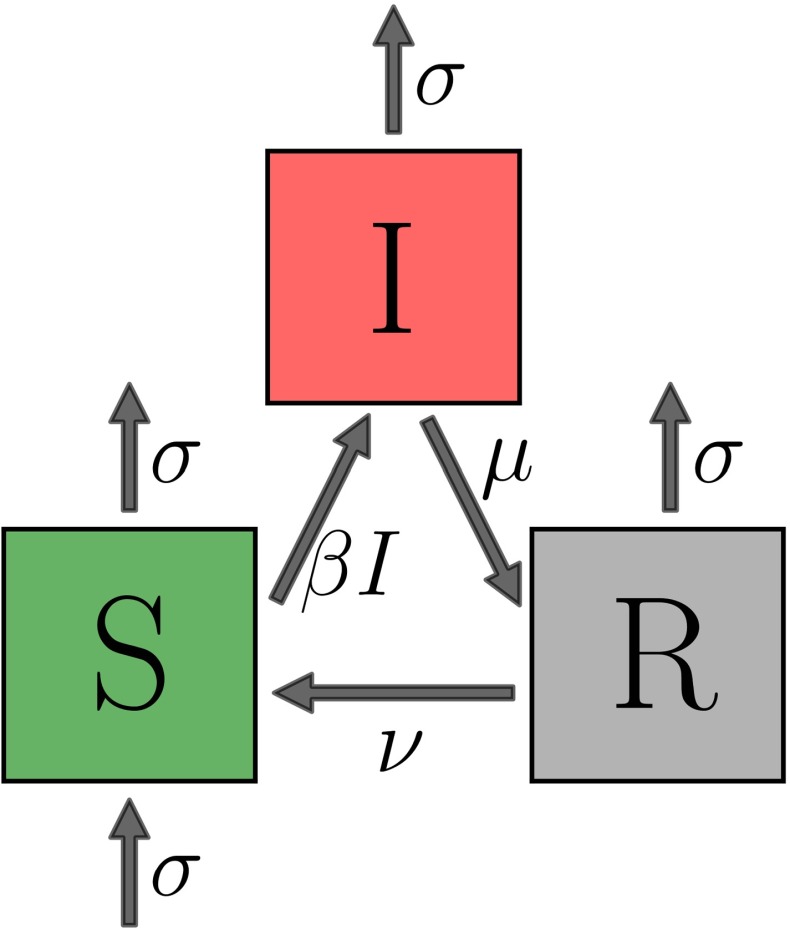
The transition structure of the SIRS compartmental model. All the rates are per host. $\beta$ is the transmission rate and therefore $\beta I$ is the rate at which susceptible hosts get infected. $\mu$ is the rate of recovery and transition to the recovered class. $\nu$ is the rate at which immunity is lost and hosts rejoin the susceptible class. Finally, $\sigma$ is both the birth and death rate, assumed to be equal

A wide range of models are used for infectious disease dynamics. Of these, many are formulated as compartmental models (Kermack and McKendrick 1927; May and Anderson 1991). The compartments represent groups of hosts who share an infection status, such as being infectious or susceptible. Considering all the hosts within one compartment as equivalent is a simplifying assumption that the transition rates between the compartments are constant that is the underlying stochastic process is Markovian. In this paper, we consider a compartmental SIRS-type model with the model structure as in Fig. 1.

This describes a situation in which the time for which the hosts stay in the infected class after infection is exponentially distributed with mean $1/\mu$. After recovery, the recovered hosts have temporary immunity and cannot be immediately reinfected. This immunity lasts for an exponentially distributed time period with mean $1/\nu$ after which the hosts rejoin the susceptible class. This model structure with temporary immunity is appropriate for diseases such as Malaria (Aron 1988; Filipe et al. 2007), Tuberculosis (Castillo-Chavez and Feng 1997) or Syphilis (Grassly et al. 2004).

We assume the population size stays constant on the time scale of the epidemic, and thus the birth rate and death rate are both equal to $\sigma$. Finally, the rate at which a susceptible host gets infected is $\beta I$, which assumes homogeneous mixing of the hosts. It can be understood as an aggregate of three terms, $n_C\times p_I\times I$ where $n_C$ is the number of contacts of an average host per unit time, $p_I$ is the probability of infection upon contact, and *I* is the proportion of infected individuals that is the probability that the contact is with an infected individual. We include a brief overview of the mathematical properties of the SIRS model in the “Appendix A1”. The effects and effectiveness of control can be introduced in a number of ways. Here we consider a treatment that can be applied to infected individuals and increases their rate of recovery by a fixed amount $\eta$ (Rowthorn et al. 2009; Ndeffo Mbah and Gilligan 2011).

To model the economical or logistic constraint, we assume that the control resources are constrained and no more than a proportion $\gamma$ of the hosts can be treated at any given time. We first analyse the deterministic version of the model, which is an approximation to the mean of the full stochastic process. The deterministic model is described by a standard set of differential equations for the proportions of susceptibles (S), infecteds (I) and removed (R), given by

1 $$\begin{aligned} \dot{I}= & {} \beta I S - (\mu +\sigma )I - \eta \min (I,\gamma ) \end{aligned}$$

2 $$\begin{aligned} \dot{R}= & {} \mu I + \eta \min (I,\gamma ) -(\nu +\sigma ) R \end{aligned}$$

3 $$\begin{aligned} S= & {} 1 - I - R. \end{aligned}$$

Here $\min (I,\gamma )$ refers to the smaller of *I* and $\gamma$. We then proceed to discuss the implications the dynamics of this model have for the stochastic behaviour. To simulate the full stochastic process, we use the standard Gillespie algorithm (Gillespie 1976).

## Model Analysis

In this section, we analyse the deterministic model (1–3) and present the complex dynamical behaviour generated by the constrained treatment term. We also show how this impacts on the stochastic dynamics of the system. To analyse the system (1–3), we calculate the fixed points and construct the bifurcation diagrams. We only consider the case when the pathogen can invade the population in the first place that is the basic reproductive number (Heffernan et al. 2005) satisfies $R_0 = \beta /(\mu +\sigma )>1$.

**Fig. 2:**
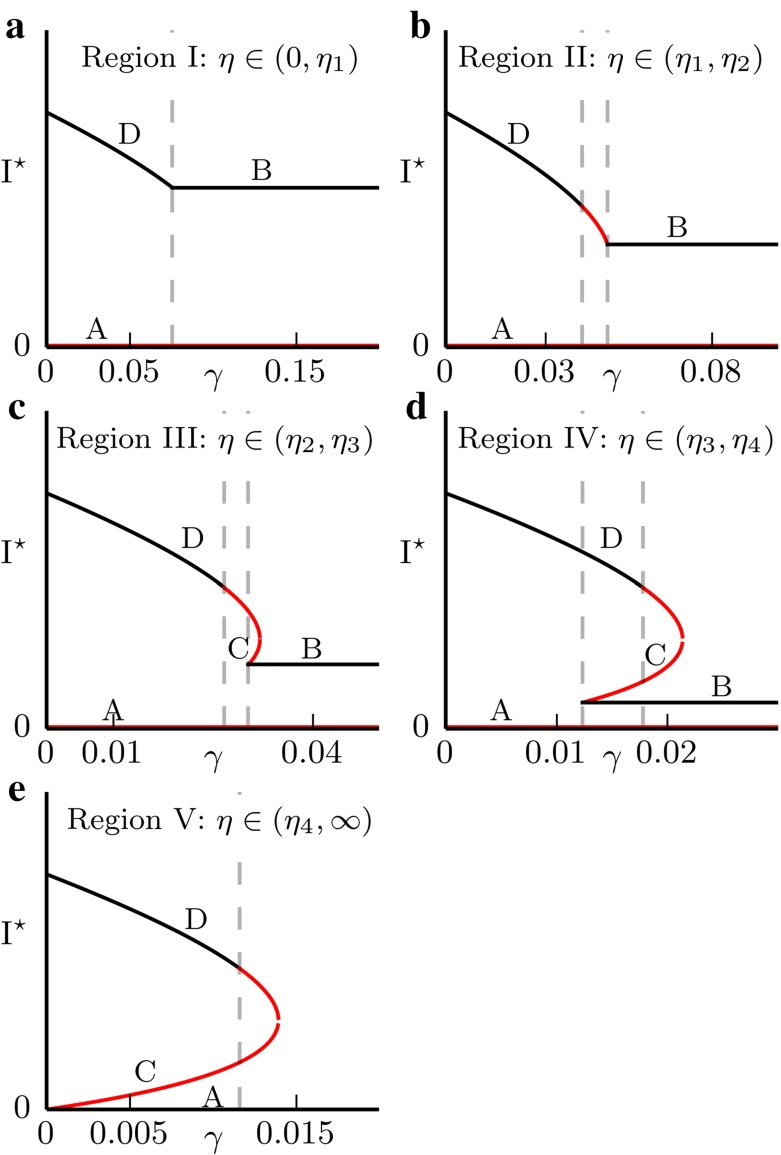
Bifurcation diagrams corresponding to the different values of $\eta$. *Black lines* mean that the fixed point is stable, *red lines* mean it is unstable. The numerical values used are; **a** $\eta =0.3$, **b** $\eta =0.65$, **c** $\eta =1$, **d** $\eta =1.5$ and **e** $\eta =2.3$. The values of the remaining parameters are $\beta =3$, $\mu =1$, $\nu =0.2$ and $\sigma =0$

For the analysis, it is useful to also define the ’full treatment’ basic reproductive number $R_0^T$ by

4 $$\begin{aligned} R_0^T = \frac{\beta }{\mu +\eta +\sigma }. \end{aligned}$$

The above system of differential equations can have at most four fixed points. There is always a fixed point at $(I,R)=(0,0)$ denoted as *A*. The point *A* is unstable when $R_0^T\ge 1$ and is stable otherwise. When *A* is stable it means that the disease can be eradicated fully if the prevalence *I* drops below a certain value. In the region $I<\gamma$, there can be another fixed point *B* given by

5 $$\begin{aligned} I_B= & {} \frac{(\nu +\sigma )\left( 1-1/R_0^T\right) }{\eta +\nu +\mu +\sigma } \end{aligned}$$

6 $$\begin{aligned} R_B= & {} \frac{(\mu +\eta )\left( 1-1/R_0^T\right) }{\eta +\nu +\mu +\sigma }. \end{aligned}$$

This fixed point is stable whenever it exists and it exists whenever $R_0^T>1$ and

7 $$\begin{aligned} \gamma >\gamma _c\equiv \frac{(\nu +\sigma )\left( 1 -1/R_0^T\right) }{\eta +\mu +\nu +\sigma }. \end{aligned}$$

This condition is simply $I_B<\gamma$. In the region $I>\gamma$, there can be two further fixed points, *C* and *D* (with $I_C<I_D$). The expressions for these fixed points are more complicated and are given in “Appendix A3”. In “Appendix A2”, Lemma 6.1, we also show that C is always a saddle point. To investigate the stability properties of D, note that as $\gamma \rightarrow 0$, D is the endemic equilibrium of the standard SIRS model without treatment and therefore it must be stable (A1). The behaviour of D as $\gamma$ increases then depends on the value of $\eta$. There are five important regions on the $\eta$ axis, I, II, III, IV resp. V, corresponding to $\eta <\eta _1$, $\eta \in (\eta _1,\eta _2)$, $\eta \in (\eta _2,\eta _3)$, $\eta \in (\eta _3,\eta _4)$ resp. $\eta >\eta _4$ (Fig. 2). The proof that the Fig. 2 is exhaustive and no other behaviour is possible, together with the analytical expressions for the critical values $\eta _i$ can be found in the “Appendix A2”, Theorem 6.2.

**Fig. 3:**
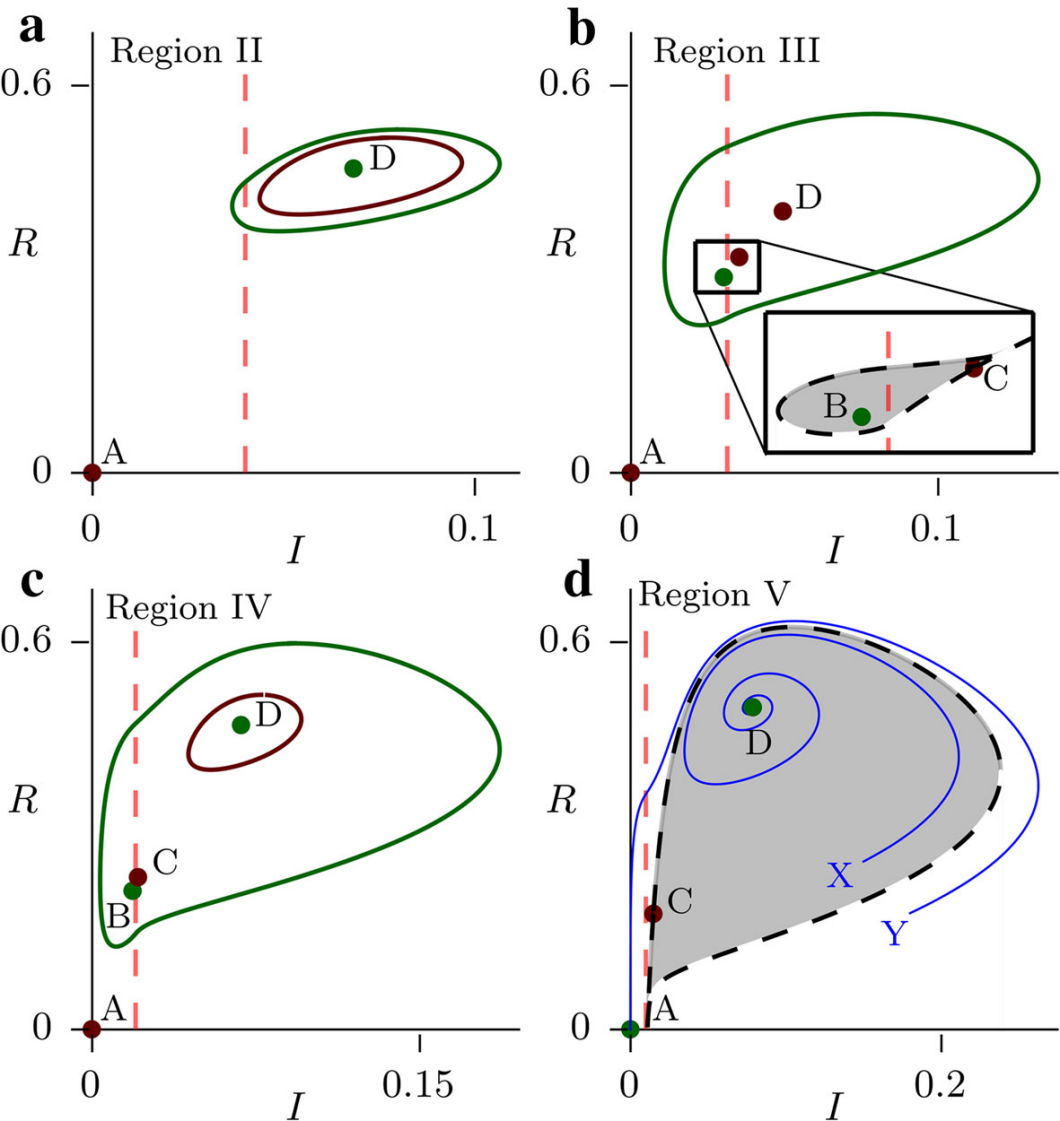
**a** The two limit cycles after they emerge through a global bifurcation around the stable fixed point D in region II. *Green* corresponds to stable, *red* to unstable. Parameter values are $\eta =0.65$, $\gamma =0.04$. **b** All four fixed points coexisting with a stable limit cycle. The detail shows the basin of attraction of B whose boundary is the stable manifold of the saddle point C. Note that the basin of attraction (*grey*) is very small and thus solutions are likely to end up on the large limit cycle. The parameter values are $\eta =1$, $\gamma =0.0315$. **c** The fixed points B and D are both stable. The behaviour is similar to that in (**b**) since the stable D together with the unstable limit cycle around it act globally as an unstable fixed point. The parameter values are $\eta =1.3$, $\gamma =0.02$. **d** Coexistence of two stable fixed points without a limit cycle. Note that the trajectory starting at *Y* leads to the disease-free state, while that starting at *X* does not, even though $I_Y>I_X$ and $R_Y<R_X$. Thus, higher initial prevalence can lead to lower long-term prevalence or even eradication. The parameter values are $\eta =2.1$, $\gamma =0.01$. In all the simulations, $\beta =3$, $\mu =1$, $\nu =0.2$ and $\sigma =0$

In region I, the bifurcation diagram is simple, with D stable throughout and continuously transitioning into B at the $\gamma =\gamma _c$ boundary (Fig. 2a). When $\eta$ increases into the region II, the fixed point D loses stability at $\gamma =\gamma _c^\prime$ before changing into B (Fig. 2b). This has implications for the phase portraits, since when D is unstable there is no stable fixed point in the system. Since the solutions are bounded, it follows from the Poincaré-Bendixson theorem that there must exist a stable limit cycle. In fact, D loses stability through a Hopf bifurcation which means there must have been an unstable limit cycle present in the system just before D became unstable. We conclude that for some unknown value or values of $\gamma <\gamma _c^\prime$, a stable and an unstable limit cycles appear in the system through global bifurcation(s). In the Fig. 3a, we show an example of the phase portrait within region II just after the two limit cycles appear in the system. As $\eta$ increases (region III, Fig. 2c), the fixed points B and C appear through a saddle-node bifurcation. D loses stability through the same Hopf bifurcation as before, and consequently there are limit cycles present. See Fig. 3b for an example of the phase portrait when all the fixed points are present in the system.

In region IV, $\gamma _c^\prime >\gamma _c$ and so D loses stability after B appears in the system. Therefore, for values of $\gamma \in (\gamma _c,\gamma _c^\prime )$ two stable endemic equilibria exist in the system. The corresponding bifurcation diagram is given in Fig. 2d. The dynamical behaviour for values of $\eta$ in the region IV is complicated and here we give an example of a phase portrait showing both B and D stable (Fig. 3c). Note that the stable limit cycle in this case is large and comes close to the $I=0$ axis. This has implications for the stochastic behaviour of the system, which are discussed in the next section, since there can be a significant probability of stochastic pathogen extinction on the limit cycle due to the very low minimal prevalence. This can happen even if the system initially starts at D, since stochastic fluctuations can perturb it outside of the unstable limit cycle.

Finally, region V corresponds to values of $\eta$ such that the fixed point A becomes stable, that is the eradication of the pathogen becomes possible. This is equivalent to $R_0^T\le 1$ and therefore $\eta _4=\beta - \mu - \sigma$. The bifurcation diagram is given in Fig. 2e and a phase portrait showing both stable D and the stable disease-free equilibrium A coexisting in Fig. 3d. Note that when two stable fixed points coexist in the system, the model predicts

a counter-intuitive dependence of the endemic equilibrium on the initial conditions (Fig. 3d). Starting the system at *X* does not achieve eradication of the pathogen, while starting it at *Y* does, even though at *Y* the prevalence is higher and the population resistance (*R*) is lower. The system also exhibits catastrophic behaviour. When $\gamma$ is increased just above $\gamma _c^\prime$, the threshold for destabilizing *D*, the system undergoes a rapid transition to the disease-free equilibrium *A*. This has obvious implications for optimal resource allocation.

**Fig. 4:**
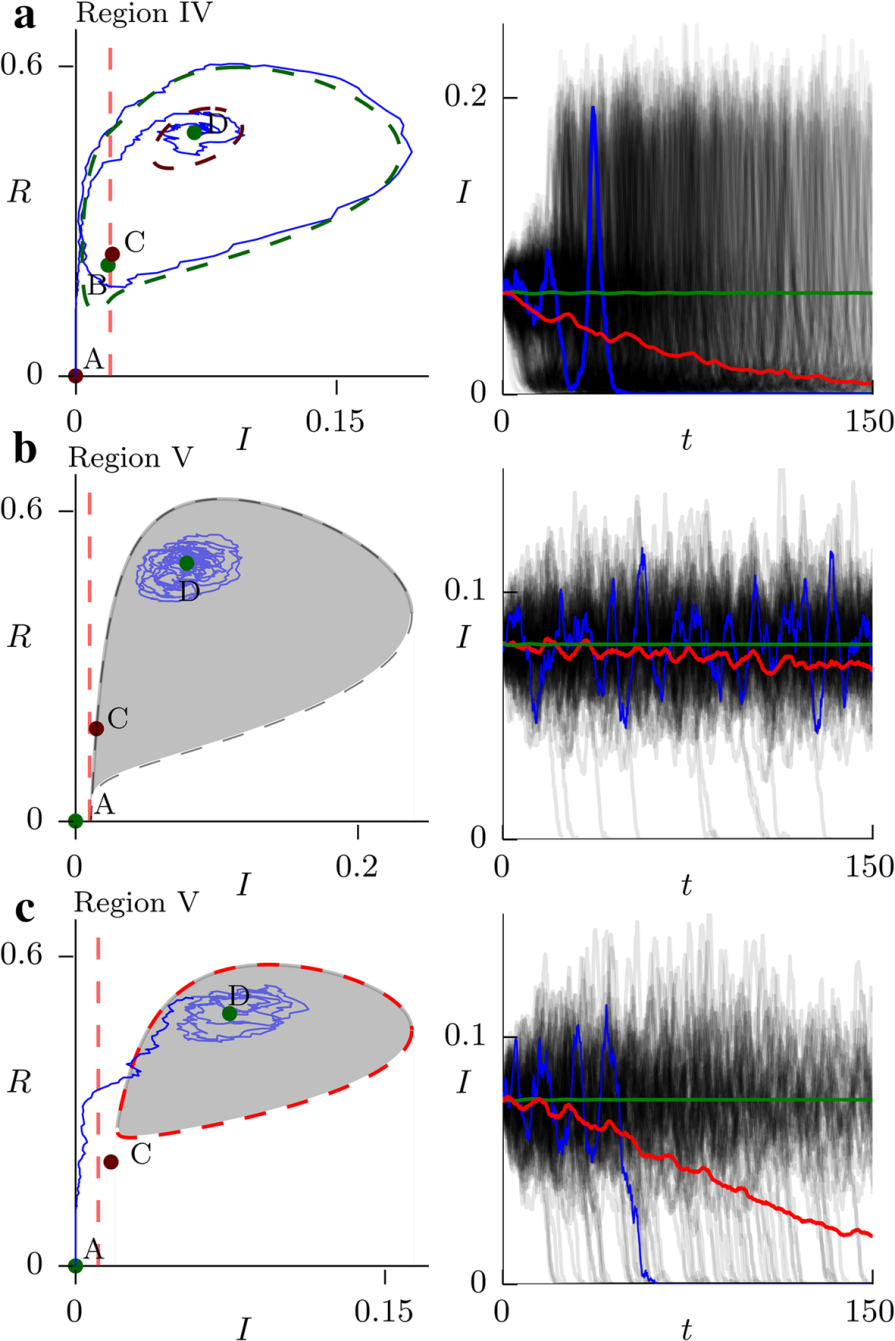
Stochastic realizations of the model in three different scenarios. The *green curve* shows the predicted deterministic behaviour, the *red curve* is the average of the stochastic realizations, and the *blue curve* shows one of the stochastic realizations. **a** The scenario from Fig. 3c. When the model is started at the stable fixed point D, the presence of the unstable limit cycle means that the stochastic fluctuations can perturb it outside and onto the large stable limit cycle. Once there, the pathogen is likely to go extinct due to the low minimum prevalence on the cycle. **b** The scenario from Fig. 3d. The trajectories fluctuate around D, but rarely go extinct, as demonstrated by the small downward slope of the *red curve*. **c** The same as b only with $\gamma$ increased from 0.01 to 0.011. The emergence of the limit cycle is enough to significantly increase the probability of extinction. In all the simulations, $\beta =3$, $\mu =1$, $\nu =0.2$, $\sigma =0$ and $N=5000$

### Stochastic Effects

The dynamical behaviour discussed in the previous section has profound consequences for the behaviour of the stochastic model. When the unstable limit cycle exists around the stable fixed point *D*, stochastic fluctuations can perturb the solution from D over the limit cycle. The system then transitions to another stable state; either a stable limit cycle or another stable fixed point. Furthermore, in regions III and IV, the stable limit cycles have large amplitude and come close to the $I=0$ axis. This means that once on the stable limit cycle, the pathogen might go extinct (Fig. 4a). The trajectories start at the fixed point *D* and fluctuate around it. Eventually, they cross the unstable limit cycle and fall onto the stable limit cycle, which leads to large amplitude oscillations. Eventually, the trajectories lead to extinction as can be seen from the downward slope of the average (red curve). This is in stark contrast to the deterministic model (green curve). It shows that when even a simple economic constraint is added, the deterministic model becomes inadequate by failing to capture the risk of extinction which can be appreciable not only when the disease prevalence is low but also in the endemic equilibrium where the disease prevalence is appreciable and where the risk of extinction would consequently be vanishing in the absence of the economic constraint.

Figure 4b, c illustrates how this impacts control. In both the system starts in the stable fixed point D. In Fig. 4b, the eradication probability is low as demonstrated by the very small downward slope of the average (red curve). The effect of increasing the resources for control, $\gamma$, by a small amount ($0.1\,\%$ of the total population) is illustrated in Fig. 4c. A global bifurcation gives rise to an unstable limit cycle around the fixed point D and consequently its basin of attraction shrinks. This significantly increases the probability of extinction, as can be seen from the steep drop in the average. This potential benefit of slightly increasing the control resources $\gamma$ would be completely hidden in the deterministic model. Thus, when deciding on the optimal value of $\gamma$ under other external constraints, such as cost of the control, it is necessary to consider the stochastic model. Relying on the deterministic model alone can lead to a gross underestimation of the effects of the control.

## Discussion

In this paper, we studied the dynamics of a simple SIRS model with treatment that increases the recovery rate of treated individuals. We considered an economic constraint on the control resources such that only a certain proportion $\gamma$ of the population can be treated at any given time. This can correspond to a limited amount of drug, insufficient infrastructure for administering the treatment or lack of specialized personnel. This model structure has been considered before in the SIRS setting. Ndeffo Mbah and Gilligan (2011) were primarily concerned with optimal allocation of drugs across two subpopulations, following early work by Rowthorn et al. (2009) who considered an SIS model. Others have previously considered optimal allocation of constrained resources in a single population (Forster and Gilligan 2007; Goldman and Lightwood 2002; Sethi and Staats 1978). Conventional analysis centres around continuously adjusting the amount of resources available to optimize the overall cost, using optimal control theory (Seierstad and Sydsaeter 1986). However, the detailed dynamics of the SIRS model with constrained control resources have not been investigated before. Since optimal control theory becomes mathematically intractably complex as more subpopulations are considered, such understanding of the dynamics of the system with constrained control is likely to be necessary for studying the more realistic problem of allocating resources between *n* interconnected populations. At the same time, the nonlinear dynamics of the constrained control system turn out to be interesting in their own right in the insight they provide on the effect of control on the inherent dynamics of the epidemic system, even without allowing for stochasticity.

We show that the system can have more than one endemic equilibrium and that the final equilibrium state which the solutions reach depends non-trivially on the initial conditions. In particular, it is possible for solutions with initially fewer infected hosts to end up in a higher-prevalence equilibrium.

The system also exhibits global bifurcations, as the critical parameter $\gamma$ (control resources) is changed, which gives rise to one or two limit cycles. The existence of the cycles has profound implications for the behaviour of the stochastic counterpart of the deterministic model. Normally, stochastic solutions initiated at a stable fixed point fluctuate around it [on time-scales shorter than exponential in *N*, the number of individuals (Allen and Burgin 2000)]. However, when there is an unstable limit cycle surrounding the fixed point, the stochastic fluctuations can perturb the solutions across the cycle. The solutions then tend to a different stable attractor. This facilitates transitions between stable attractors which would be much less likely in a stochastic system without the unstable limit cycle and would not be possible at all in the deterministic system. This is of particular importance when one of the attractors in question is the disease-free equilibrium because the combined effect of the stochastic fluctuations and the deterministic dynamics might then facilitate disease eradication. This is a benefit of the control deployment which would not be revealed if the detailed dynamics were not considered. Furthermore, the stable limit cycle often comes very close to the $I=0$ axis and thus may facilitate stochastic extinction of the pathogen even if the disease-free equilibrium of the deterministic system is not stable.

We conclude that when an external constraint on the control resources is imposed, stochastic effects together with the detailed dynamics of the system must be considered in order to understand the range of potential effects the control may have. Neglecting this may lead to underestimation of the positive impact of the control and therefore to wasting resources by overallocation or by incorrectly deciding not to apply control at all. Note that most of the non-trivial dynamical behaviour is occurring close to $\gamma =\gamma _c$, which for the parameter values considered in this paper corresponds to the ability of treating between 1 and 8 % of the population simultaneously at any given time. These values are low but plausible in situations where the proportion of individuals that can be treated is limited by the shortage of infrastructure or personnel to administer the control. Furthermore, when designing an optimal control coverage (optimal value of $\gamma$), selecting $\gamma$ high above the critical threshold $\gamma _c$ leads to the number of the infected individuals in the endemic state being much smaller than the number that can be treated, that is, resources will be wasted. This means that the optimal value of $\gamma$ is expected to be close to $\gamma _c$ and thus in the region where the non-trivial dynamics are important.

There are several directions in which this work could be taken forward. First is an extension of the rigorous analysis to investigate whether the complex dynamics and the qualitative differences between the deterministic and stochastic behaviour are present when the effects of control are modelled differently or when disease-induced death occurs. As an example of the former, the control can be modelled to reduce the infectiousness of the controlled individuals via hospitalization or quarantine. Further to this, in models where qualitative differences occur between deterministic and stochastic versions, the parameter space could be scanned in its entirety to get a measure of how often the divergence is sufficiently large to be significant in considering the effectiveness of control programmes. Second is to consider the practical applications of the understanding developed in this work in the context of the problem of how to optimally allocate limited resources within a network of *n* interconnected populations.

## Appendix

### Dynamics of the Standard SIRS Model

A full description of the dynamical behaviour of the SIRS model can be found in most standard textbooks, for example Keeling and Rohani (2008) or May and Anderson (1991). Here we list the most important properties for convenience. The SIRS model at its most basic is described by the equations:

8 $$\begin{aligned} \dot{S}= & {} \sigma + \nu R - \sigma S - \beta I S \end{aligned}$$

9 $$\begin{aligned} \dot{I}= & {} \beta I S - (\mu + \sigma )I \end{aligned}$$

10 $$\begin{aligned} \dot{R}= & {} \mu I - (\nu + \sigma )R. \end{aligned}$$

Here *S*, *I* and *R* and the proportions of the susceptible, infectious and recovered individuals, respectively. $\beta$ is the transmission rate of the infection, $\sigma$ is the birth rate and the death rate, which are assumed to be equal, $\mu$ is the recovery rate and $\nu$ is the rate with which recovered individuals lose immunity and rejoin the susceptible class.

The system has one or two equilibria. One is the disease-free equilibrium $I^\star =0$, $R^\star =0$ and $S^\star =1$. The other, whenever it exists, is an endemic equilibrium given by:

11 $$\begin{aligned} S^\star= & {} \frac{1}{R_0} \end{aligned}$$

12 $$\begin{aligned} I^\star= & {} \frac{\nu +\sigma }{\nu +\sigma +\mu }\left( 1-\frac{1}{R_0}\right) \end{aligned}$$

13 $$\begin{aligned} R^\star= & {} \frac{\mu }{\nu +\sigma +\mu }\left( 1-\frac{1}{R_0}\right) , \end{aligned}$$

where we have defined the so-called basic reproductive number $R_0=\beta /(\mu +\sigma )$. The basic reproductive number measures on average how many new infections an infectious individual will cause before recovering. When $R_0\le 1$, an epidemic is impossible, the disease-free equilibrium is stable and the endemic equilibrium does not exist. When $R_0>1$, the disease-free equilibrium is unstable and the endemic equilibrium is a stable global attractor.

### Proofs of the Key Results

#### Lemma 4.1

C is a saddle whenever it exists.

#### Proof

First, it is straightforward to find the expressions for the fixed points C and D. They are given by

14 $$\begin{aligned} I_C= & {} \frac{\chi -\sqrt{\chi ^2 -P}}{2\beta (\mu +\nu +\sigma )} \end{aligned}$$

15 $$\begin{aligned} I_D= & {} \frac{\chi +\sqrt{\chi ^2 -P}}{2\beta (\mu +\nu +\sigma )} \end{aligned}$$

16 $$\begin{aligned} R_{C,D}= & {} \frac{\mu I_{C,D} + \gamma \eta }{\nu +\sigma } \end{aligned}$$

where

17 $$\begin{aligned} \chi= & {} \beta (-\gamma \eta +\nu +\sigma )-(\mu +\sigma )(\nu +\sigma ) \end{aligned}$$

18 $$\begin{aligned} P= & {} 4\beta \gamma \eta (\nu +\sigma )(\mu +\nu +\sigma )>0. \end{aligned}$$

When $I>\gamma$, the Jacobian of the system is

19 $$\begin{aligned} J(I,R) = \left( \begin{array}{cc} \beta (1-R-2I)-(\mu +\sigma ) &{} -\beta I \\ \mu &{} -(\nu +\sigma ) \end{array} \right) . \end{aligned}$$

From this, we can calculate the determinant evaluated at the point $(I_C,R_C)$. It is given by

20 $$\begin{aligned} \det J_C= & {} -(\beta (1-R_C-2I_C)-(\mu +\sigma ))(\nu +\sigma ) + \beta \mu I_C \end{aligned}$$

21 $$\begin{aligned}= & {} (\mu +\sigma )(\nu +\sigma )-\beta \left( \nu +\sigma -\gamma \eta -\frac{1}{\beta }(\chi -\sqrt{\chi ^2-P})\right) \end{aligned}$$

22 $$\begin{aligned}= & {} -\sqrt{\chi ^2 - P} < 0 \end{aligned}$$

$$\square $$

#### Theorem 4.2

The bifurcation diagrams in Fig. 2 cover all the possible behaviour of the fixed points.

#### Proof

We have discussed stability of A and B in the main text and in lemma 6.1 we proved that C is always a saddle. To prove this theorem, we first consider what happens to D as $\gamma$ increases. $I_D$ must be a decreasing function of $\gamma$. What is its value when $\gamma =\gamma _c$? Inserting the expression for $\gamma _c$ into the formula for $I_D$ gives that

23 $$\begin{aligned} I_D(\gamma _c) = {\left\{ \begin{array}{ll} I_B &{} \text{ if } \eta \le \eta _2 \\ \frac{\eta (\nu +\sigma )}{\beta (\nu +\mu +\sigma )}> I_B &{} \text{ if } \eta > \eta _2. \end{array}\right. } \end{aligned}$$

where

24 $$\begin{aligned} \eta _2 = -(\mu +\nu +\sigma ) + \sqrt{(\mu +\nu +\sigma )(\beta +\nu )}. \end{aligned}$$

Note this is the $\eta _2$ that separates regions II and III on the $\eta$ axis, in Fig. 2. This is the value of $\eta$ at which the transition from D to B at $\gamma =\gamma _c$ becomes discontinuous. The argument presented here constitutes a derivation of its value. Now, at $\gamma =0$, D is stable. As $\gamma$ increases, Tr$J_D > 0$ is equivalent to $\gamma \in (\gamma _c^\prime , \gamma _0)$ where $\gamma _c^\prime$ and $\gamma _0$ are the roots of the corresponding quadratic equation. So D can change its stability properties at most twice. For the purposes of the analysis here, only $\gamma _c^\prime$ will be needed. It is given by

25 $$\begin{aligned} \gamma _c^\prime= & {} \frac{1}{2\beta \eta }\left( \mu ^2 + 3\mu (\nu +\sigma ) + 2(\nu +\sigma )(\beta +2\nu +\sigma )-(\mu +2\nu +2\sigma ) \right. \nonumber \\&\left. \sqrt{(\mu +\nu +\sigma )^2+4(\nu +\sigma )(\beta +\nu )}\right) . \end{aligned}$$

Now we need to check when D loses stability and whether it happens for $\gamma <\gamma _c$. Solving the quadratic inequality $\gamma _c^\prime >\gamma _c$, which, when satisfied, means that at $\gamma _c$, D is still stable, gives $\eta \not \in (\eta _1,\eta _3)$ where $\eta _1$ and $\eta _3$ are the roots of the corresponding quadratic equation, given by

26 $$\begin{aligned} \eta _1= & {} \frac{1}{2}\left( -\mu -\nu -\sigma +\sqrt{(\mu +\nu +\sigma )^2+4(\nu +\sigma )(\beta +\nu )}\right) \end{aligned}$$

27 $$\begin{aligned} \eta _3= & {} \frac{1}{2(\nu +\sigma )}\left( -(\mu +\nu +\sigma )(\mu +3\nu +3\sigma ) \right. \nonumber \\&\left. +\sqrt{(\mu +\nu +\sigma )^2+4(\nu +\sigma )(\beta +\nu )}\right) . \end{aligned}$$

These are the critical values separating the regions I and II resp. III and IV on the $\eta$ axis, in the Fig. 2. This means that for $\eta <\eta _1$, D stays stable until it continuously transitions into B (see Fig. 2a). Now we are ready to consider the other cases in turn:

- The case $\eta \in (\eta _1,\eta _2)$. In this case, C cannot exist because D transitions into B continuously. We will show that it is impossible for D to lose stability and then become stable again before $\gamma$ reaches $\gamma _c$. To do this we consider the sign of $\hbox {Tr}J_D$ at $\gamma =\gamma _c$, when $I_D(\gamma _c)=I_B$. This is equal to
  28 $$\begin{aligned} \text {Tr}J_D = \frac{\eta ^2-(\beta +\nu )(\nu +\sigma )+\eta (\mu +\nu +\sigma )}{\eta +\mu +\nu +\sigma } \end{aligned}$$
  Setting $\hbox {Tr}J_D<0$ then gives a quadratic inequality which is satisfied if and only if $\eta <\eta _1$. Therefore, when $\eta \in (\eta _1,\eta _2)$ the only possible behaviour is such that D loses stability for some $\gamma <\gamma _c$ and then continuously transitions into B. This justifies Fig. 2b.
- The case $\eta \in (\eta _2,\eta _4)$. We already know that in this case, $I_D>I_B$ at $\gamma =\gamma _c$. Now we will show that this means C always appears at $\gamma =\gamma _c$. For C to exist, we must have $I_C>\gamma$. This is once again a quadratic inequality and holds whenever $\gamma \in (\gamma _c,\gamma _b)$ where the root $\gamma _b$ is given by
  29 $$\begin{aligned} \gamma _b = \frac{(\beta -\mu -\sigma )(\nu +\sigma )}{\beta (\eta +2(\mu +\nu +\sigma ))}. \end{aligned}$$
  When does this interval exist? Setting $\gamma _b>\gamma _c$ and solving for $\eta$ reveal that the interval does exist when $\eta >\eta _2$. This proves that for $\eta >\eta _2$, C appears at $\gamma =\gamma _c$. As $\gamma$ increases further, there are two ways C can cease to exist. Either $\gamma$ reaches $\gamma _b$ or C and D collide, which happens when $\chi ^2=P$. Solving this quadratic equation for $\gamma$ reveals that this first happens at $\gamma _a$ given by
  30 $$\begin{aligned} \gamma _a = \frac{\nu +\sigma }{\beta \eta }\left( \beta +\mu +2\nu +\sigma -2\sqrt{(\mu +\nu +\sigma )(\beta +\nu )}\right) . \end{aligned}$$
  We will now show that $\gamma _b>\gamma _a$ which means that C and D always collide and annihilate. We want to show that
  31 $$\begin{aligned} \frac{\nu +\sigma }{\beta \eta }\left( \beta +\mu +2\nu +\sigma -2\sqrt{(\mu +\nu +\sigma )(\beta +\nu )}\right) <\frac{(\beta -\mu -\sigma )(\nu +\sigma )}{\beta (\eta +2(\mu +\nu +\sigma ))}. \end{aligned}$$
  After solving for $\eta$, this simplifies to $\eta > (\nu +\mu +\sigma )(\beta +\nu -\eta _2)/\eta _2$. We have divided by $\eta _2$ because it is always positive, as can be quickly checked using the assumption $\beta >\mu +\sigma$. Simple algebra reveals that in fact $(\nu +\mu +\sigma )(\beta +\nu -\eta _2)/\eta _2=\eta _2$ and therefore the above inequality reduces to $\eta >\eta _2$ which is trivially satisfied by assumption. To finish the justification of the bifurcation diagrams in Fig. 2c, d, we need to check that when C and D collide, D is always unstable. To do this we need to show that $\gamma _c^\prime <\gamma _a$ and a proof of this is the subject of Lemma 6.3.
- The final case, $\eta >\eta _4$. This region corresponds to $R_0^T\ge 1$ and therefore $\eta _4=\beta -\mu -\sigma$. Since the disease can be eradicated, the point A is stable. The behaviour of D and C does not change compared to the case $\eta \in (\eta _2,\eta _4)$. They will be present as long as $\gamma _a>0$ and for $\beta >\mu +\sigma$ this is always the case.

$$\square $$

#### Lemma 4.3

$$\gamma _c^\prime <\gamma _a$$.

#### Proof

Consider the quantity $D=2\beta \eta (\gamma _c^\prime -\gamma _a)$. Define new variables $x=\beta +\nu$ and $y=\sigma +\nu$ and without loss of generality, set $\mu =1$ (this is just a rescaling of time). Then we can write

32 $$\begin{aligned} D(x,y) = 2y\sqrt{x(1+y)}+1+y-(1+2y)\sqrt{1+2y+y(4x+y)}, \end{aligned}$$

where $x>1$ and $y>0$. We will show that *D* is always negative. After squaring $D<0$ reads

33 $$\begin{aligned} 4y^2x(1+y)+(1+y)^2+4(1+y)y\sqrt{x(1+y)}<(1+2y)^2(1+2y+y^2+4xy). \end{aligned}$$

Separating the square root and squaring again gives

34 $$\begin{aligned} x(1+y)^3 < [(1+y)^3 + x(1+3y+3y^2)]^2 \end{aligned}$$

and rearranging the terms leads to

35 $$\begin{aligned} x^2(1+3y+3y^2)^2+(1+y)^3(1+6y+6y^2)x+(1+y)^6>0, \end{aligned}$$

which is evidently satisfied. This finishes the proof. $\square$

## References

[CR1] Allen LJS, Burgin AM (2000). Comparison of deterministic and stochastic SIS and SIR models in discrete time. Math Biosci.

[CR2] Aron JL (1988). Mathematical modelling of immunity to malaria. Math Biosci.

[CR3] Castillo-Chavez C, Feng Z (1997). To treat or not to treat: the case of tuberculosis. J Math Biol.

[CR4] Filipe JAN, Riley EM, Drakeley CJ, Sutherland CJ, Ghani AC (2007). Determination of the processes driving the acquisition of immunity to malaria using a mathematical transmission model. Plos Comput Biolo.

[CR5] Forster GA, Gilligan CA (2007). Optimizing the control of disease infestations at the landscape scale. Proc Natl Acad Sci.

[CR6] Geoffard P, Philipson T (1996). Rational epidemics and their public control. Int Econ Rev.

[CR7] Gillespie DT (1976). A general method for numerically simulating the stochastic time evolution of coupled chemical reactions. J Comput Phys.

[CR8] Goldman SM, Lightwood J (2002) Cost optimization in the SIS model of infectious disease with treatment. Top Econ Anal Policy 2(1)

[CR9] Grassly NC, Fraser C, Garnett GP (2004). Host immunity and synchronized epidemics of syphilis across the United States. Nature.

[CR10] Hastings A (2001). Transient dynamics and persistence of ecological systems. Ecolo Lett.

[CR11] Heffernan JM, Smith JR, Wahl LM (2005). Perspectives on the basic reproductive ratio. J R Soc Interface.

[CR12] Hethcote HW, Levin SA (1989). Periodicity in epidemiological models. Appl Math Ecol.

[CR13] Jin Y, Wang W, Xiao S (2007). An SIRS model with a nonlinear incidence rate. Chaos Solitons Fractals.

[CR14] Kaung Y, Freedman HI (1988). Uniqueness of limit cycles in Gause-type models of predator-prey systems. Math Biosci.

[CR15] Keeling MJ, Rohani P (2008). Modeling infectious diseases in humans and animals.

[CR16] Keeling MJ, Rohani P, Grenfell BT (2001). Seasonally forced disease dynamics explored as switching between attractors. Phys D Nonlinear Phenom.

[CR17] Kermack WO, McKendrick AG (1927). A contribution to the mathematical theory of epidemics. Proc R Soc A.

[CR18] Klein E, Laxminarayan R, Smith DL, Gilligan CA (2007). Economic incentives and mathematical models of disease. Environ Dev Econ.

[CR19] May RM, Anderson RM (1991). Infectious diseases of humans: dynamics and control.

[CR20] Ndeffo Mbah ML, Gilligan CA (2010). Balancing detection and eradication for control of epidemics: sudden oak death in mixed-species stands. Plos One.

[CR21] Ndeffo Mbah ML, Gilligan CA (2011). Resource allocation for epidemic control in metapopulations. Plos One.

[CR22] Rowthorn RE, Laxminarayan R, Gilligan CA (2009). Optimal control of epidemics in metapopulations. J R Soc Interface.

[CR23] Seierstad A, Sydsaeter K (1986). Optimal control theory with economic applications.

[CR24] Sethi SP, Staats PW (1978). Optimal control of some simple deterministic epidemic models. J Oper Res Soc.

[CR25] Toupo DFP, Strogatz SH (2015). Nonlinear dynamics of the rock-paper-scissors game with mutations. Phys Rev E.

[CR26] Wang W, Ruan S (2004). Bifurcations in an epidemic model with constant removal rate of the infectives. J Math Anal Appl.

